# Strengthening neonatal care through ward assistants: a Kenyan case study in enhancing infection prevention and control practices

**DOI:** 10.1186/s13756-025-01575-w

**Published:** 2025-06-02

**Authors:** Michuki Maina, Nancy Odinga, Vincent Kagonya, Gloria Ngaiza, Sebastian Fuller, Onesmus Onyango, Caroline Waithira, Dorothy Oluoch, David Gathara, Peter Mwangi, Loise Mwangi, Penina Musyoka, Lucy Kinyua, Lydia Thuranira, Virginia Njoroge, Ngina Mwangi, Zainab Kioni, Mike English, Edna Mutua

**Affiliations:** 1 KEMRI-Wellcome Trust Research Programme, Health Services Research Group, PO Box 43640-00100, Nairobi, Kenya; 2 Nuffield Department of Medicine, The University of Oxford, Peter Medawar Building for Pathogen Research, South Parks Road, Oxford, OX1 3SY UK; 3 London School of Hygiene and Tropical Medicine, Keppel St, London, WC1E 7HT UK; 4 Department of Health, Nyeri County, P.O. Box 1112-10100, Nyeri, Kenya; 5 Department of Health, Embu County, P.O. Box 36-60100, Embu, Kenya; 6 Department of Health, Machakos County, P.O. BOX 1996, Machakos, 90100 Kenya; 7 Department of Health, Kiambu County, P.O. Box 2344-00900, Kiambu, Kenya

**Keywords:** Infection prevention, Ward assistants, Neonatal care, Health workforce, Quality of care

## Abstract

**Background:**

Infection prevention and control (IPC) is a critical component of neonatal care, particularly in low- and middle-income countries (LMICs), where healthcare settings face unique challenges. Neonates, especially preterm and low birth weight infants, are at higher risk for infections, including healthcare-associated infections. In Kenya, neonatal units struggle with limited resources, understaffing, and shortages of essential supplies, significantly impeding effective IPC practices.

**Methods:**

This study employed a mixed methods approach in four public neonatal units in Kenya to assess the impact of deploying ward assistants on IPC practices. Data collection included structured and unstructured observations, in-depth interviews, and focus group discussions with healthcare workers and caregivers. The intervention aimed to address gaps in routine cleanliness, waste management, and adherence to IPC protocols.

**Results:**

The introduction of ward assistants led to noticeable improvements in overall ward cleanliness and waste disposal, highlighting the potential for enhanced infection control. Mothers’ hand hygiene practices improved, driven by targeted sensitization efforts. Despite these gains, significant challenges remained. Hand hygiene adherence among healthcare providers was inconsistent, and equipment cleaning and decontamination were frequently compromised by insufficient supplies and overwhelming patient demand. The findings underscored the critical role of resources and the need for consistent supervision and training to support sustainable IPC improvements.

**Conclusion:**

Deploying ward assistants in neonatal units can positively influence IPC practices, particularly in addressing environmental cleanliness and waste management. However, these benefits alone are insufficient to address systemic barriers to IPC, including resource constraints and variability in adherence among staff. To sustain these gains, robust training, consistent supervision, and adequate resourcing are imperative. Future research should explore the long-term impact of such interventions and design context-specific strategies to overcome persistent barriers, ensuring safer neonatal care in resource-limited settings.

**Clinical trial number:**

Not applicable.

**Supplementary Information:**

The online version contains supplementary material available at 10.1186/s13756-025-01575-w.

## Background

It is estimated that 2.4 million newborns die each year, with a substantial proportion due to infections [1]. Mortality within neonatal units is also high; in Kenya, for example, a crude mortality rate of 10% was noted among inborn neonates admitted to the neonatal units (NBU) [2]. Neonates who are admitted into these units and require longer hospital stays, especially preterm and low birth-weight infants, whose immature immune systems make them highly susceptible to infections, are at higher risk of healthcare-associated infections (HCAI) [2, 3]. Infection prevention and control (IPC) therefore forms a critical component of healthcare provision in these neonatal units. The neonatal units in many low- and middle-income countries (LMICs) face specific challenges that make it difficult to comply with the required IPC standards. These include shortages of water, infrastructure, and supplies [4].

To improve care, there have been global efforts to equip neonatal units with essential technologies. One such program is the Neonatal Essential Technologies (NEST 360) program which has added equipment including radiant warmers, incubators, phototherapy devices, and respiratory support systems to 13 NBUs in Kenyan public hospitals [5]. However, technologies themselves pose IPC challenges and can contribute to HCAI. The introduction of such life-saving equipment therefore needs a correspondingly robust IPC program [6, 7].

Many neonatal units in Kenya, especially those within public hospitals, still face the challenge of extreme patient-nurse ratios, with some as low as one nurse to 40 babies and often generally poor infrastructure [8]. These low staffing ratios see some crucial tasks, including IPC-related tasks, being missed or left undone. This may affect patient safety and lead to poor outcomes [8, 9]. IPC in many neonatal units is thus undermined by poor infrastructure, poor staffing, and by deployment of technologies in settings where maintenance of these technologies is poorly supported. At the same time, the patient population is increasingly made up of preterm / very low birth weight babies with heightened vulnerability.

From the health worker perspective, it has been estimated that nurses in NBUs spend almost a quarter of their nursing time on non-direct care activities including environmental cleaning and decontamination, waste disposal and supervision of mothers and caregivers on essential IPC tasks [10]. By delegating these responsibilities to trained non-clinical staff, nurses can dedicate more time to direct patient care activities [11]. These models have been shown to streamline workflows and increase efficiency with improved patient outcomes [12].

Here we examine IPC practices in four neonatal units in Kenyan public hospitals that were recipients of NEST 360 technologies using a mixed methods approach. Additionally, we explore the potential value of ward assistants to promote and improve IPC in these clinical contexts.

## Methods

This study was embedded in a larger pre-post study that examined the changes in the quality of care after the introduction of additional nurses and ward assistants in facilities [13, 14]. In summary, the study was conducted in four public neonatal units in Kenya. The four facilities are part of 13 hospitals that received a bundle of medical technologies in 2021 that included radiant warmers, Continuous Positive Airway Pressure machines (CPAP), phototherapy machines and oxygen concentrators under the NEST 360 programme [5]. These units aim to provide intermediate-level neonatal care led by one to two paediatricians, with medical officers, non-physician clinicians and nurses. These facilities are also training centres for diploma-level nurses and internship centres for doctors and clinicians. A detailed table on the hospital characteristics and staffing numbers if provided as a supplement (Appendix 1).

### Data collection

#### Facility assessment using checklists

The first category of data was collected using a facility assessment of key IPC infrastructure and status. These assessments were conducted by two research assistants (VK and OO) who are trained nurses with knowledge and experience of care procedures and standards in Kenyan hospitals. The assessments were carried out over a total of 27 randomly selected 12-hour shifts across the facilities covering both weekdays and weekends (including night shifts). These data collection tools were piloted before the study commenced and any necessary improvements were made to promote reliability. The tools captured IPC-related activities that were carried out in the unit across four domains (Table 1 and Appendix 2). For the hand hygiene domains, the research assistants observed instances where mothers, nurses, clinicians, and students practised hand hygiene. For the clinicians, nurses, and students, hand hygiene was evaluated against the World Health Organization (WHO) five moments of hand hygiene [15]. For the mothers, critical moments where hand hygiene is expected were assessed in this case, four main moments were assessed, these were; Hand hygiene assessed before holding the baby, before breastfeeding or feeding the baby, after changing diapers and after touching the hospital surfaces or equipment. There was no set minimum number of observations for the hand hygiene events but based on their observation notes the assessor assigned an overall shift-level aggregate score at the end of the shift for hand hygiene practices (Table 1). In the same way, the observers kept notes on other IPC practices within the shift, and at the end of the shift assigned an overall shift-level aggregate score against each criterion defined in Table 1.

**Table 1:** IPC domains, indicators assessment metric

Domain	Indicators/Practices assessed	Assessment Metric
**Hand hygiene practices and supplies**	Mothers/Caregivers practice hand hygiene. (Hand hygiene assessed before holding the baby, before breastfeeding, after changing diapers or after touching the hospital surfaces)	Likert scale: Never, rarely, occasionally, often, always
	Clinicians, nurses, and students practice the WHO five moments of hand hygiene; (Before touching a patient, before a procedure, after a procedure or body fluid exposure risk, after touching a patient, after touching a patient’s surroundings.	Likert scale: Never, rarely, occasionally, often, always
	There is a continuous supply of water, soap, hand towels, and alcohol hand rubs.	No, yes
	Sinks for handwashing are available, clean, and functional	No, yes
	There are posters/charts on handwashing in the ward or hand washing stations	No, yes
**General ward cleanliness**	The surfaces, walls and floors are kept clean with no visible marks or dirt.	No, yes
	The cleanliness status of the of the beds and cots	No, yes
	The cleanliness and changing of bed linen & mothers’ gowns	No, yes
	The adequacy and cleanliness of footwear for both staff and mothers	No, yes
**Equipment cleaning and decontamination**	Nasal prongs, incubators, oxygen concentrators, pulse oximeters, radiant warmers, phototherapy, and feeding cups are consistently kept clean and routinely decontaminated	No, yes
	Cleaning rotas and equipment cleaning instructions are available in the unit	No, yes
**Waste management**	Colour-coded waste disposal bins with liners and sharps disposal containers are available and functional	No, yes
	Waste is correctly segregated into appropriately coloured bins.	No, yes
	Pictures/posters of waste segregation are available next to the waste collection bins	No, yes

#### Non-structured observations, interviews and focus group discussions (FGDs)

In addition to the structured assessments conducted by the research assistants, non-participant observations were conducted by two non-clinician observers (NO, CW), social scientists with no previous experience working in a hospital setting. They employed an ethnographic approach and were stationed in the neonatal units over extended periods to generate data to examine neonatal care quality more broadly. As part of this, they documented in ‘field notes’ ward sanitation and hygiene practices, interactions among mothers, clinicians, and ward assistants, and IPC counselling practices. Additionally, they conducted interviews with nurses and FGDs with groups of six to nine mothers. The semi-structured interview guide, which was for the larger project but included specific questions on IPC, was developed through informal discussions with stakeholders and clinicians and then refined iteratively based on observations and initial interview findings. Interviews were expected to last between 30 and 90 minutes and were audio-recorded in quiet hospital areas. FGDs provided insights into mothers’ experiences and IPC-related practices.

### Data analysis

#### Facility assessment data

For facility assessment data, binary outcome (yes or no) assessments were made for the structural and supplies indicators and given a numeric score of 1 or 0 respectively. Observed behaviours and practices were scored using a 5-point Likert scale (Table 1) and converted to numeric scores (0 -Never, 0.25 -Rarely, 0.5- Occasionally, 0.75 -Often, 1 -Always). Aggregate scores were calculated by summing the numeric scores as a proportion of the total possible score for each of the domains assessed in each of the facilities during the two rounds of assessment. Later, descriptive statistics were generated, presented, and reported using simple histograms/bar charts. The analysis was conducted in R statistical software [16].

#### Qualitative data

The field notes were entered into NVIVO 14 software for inductive coding and thematic analysis with a focus on examining IPC and hand hygiene activities, behaviours, communications, and relationships. For the audio-recorded interview and FGD data, the audio files were kept on an encrypted laptop. These were transcribed and uploaded into NVivo 14 software [17]. NO, and CW coded the transcripts independently before discussing the codes with DO and EM and agreeing on combined axial codes that highlight key information about the IPC arrangements in the four hospitals. The findings from the interviews are presented as quotes to illustrate some of the insights gained. The semi structured interview guides are provided as a supplement (Appendix 3).

#### Data integration

For this mixed methods approach, the analysis of data from the facility assessment and the qualitative data were done separately. The results were brought together with the qualitative data explaining some of the facility assessment data.

### Ward assistants intervention

As part of the parent study each of the four study hospitals received an additional three ward assistants for seven months in the study period [13]. The ward assistants were recruited and trained for 1 week in different non-clinical tasks including IPC tasks like ward cleaning, waste management device cleaning and effective communication. They were supervised by the ward managers and were provided with a clear job description (Appendix 4). Table 2 below shows the number of ward assistants before and after the intervention, their allocation across shifts and IPC duties covered in the wards. The low numbers per shift was attributed to those on leave or off duty. However, during the study period, there was redeployment of the resident ward assistants to other units within the hospital. In H3, one intervention ward assistant resigned, and one resident ward assistant was redeployed elsewhere during the intervention period. Also, for H3 there is no ward assistant for night shift - from May - July 2023. In H4, one resident ward assistant from was redeployed elsewhere two months after the intervention. The Ward assistants’ intervention is described in more detail elsewhere [18].

**Table 2:** Ward assistant distribution and roles within the facilities

Hospital		H1		H2		H3		H4	
Study Phase		Pre intervention	Post intervention	Pre intervention	Post intervention	Pre intervention	Post intervention	Pre intervention	Post intervention
Total Ward Assistants		**3**	**6**	**3**	**6**	**1**	**4**	**2**	**5**
Distribution of ward assistants per shift	**Day**	**1**	**3**	**2**	**3**	**1**	**1**	**1**	**1**
	**Night**	**1**	**1**	**1**	**1**	**0**	**1**	**1**	**1**

**IPC-specific Ward Assistant Roles**

**Routine newborn care**: Linen and diaper change, baby cleaning and assisting mothers with cord care

**Device and equipment cleaning**: Cleaning and preparing feeding utensils, equipment cleaning (including decontamination

**General cleaning and waste management**: cleaning floors, surfaces, and waste management (lining bins, emptying bins, setting up sharps boxes

**Mother orientation**: Orienting new mothers on ward set up including hand hygiene and waste disposal stations

## Results

Here we first describe the facilities and IPC practices generally and then proceed to describe the effects of the ward assistants on IPC.

The four included NBU facilities are in urban and semi-urban regions with an average of about 34 babies and 2–3 nurses per shift over the study period. The first round of data collection was pre intervention. This commenced in March 2022, with the hospital assessments by the research assistants taking place over a month until April 2022. The nonparticipant observations also commenced at that time and progressed over a duration of sixteen months as part of the larger project. The ward assistants were introduced in January 2023 for a duration of seven months. During this period the nonparticipant observations and interviews were ongoing. The second round of hospital assessments (post ward assistant intervention) were carried out in June and July of 2023 this was six months after the ward assistant’s introduction.

We observed a total of 27, 12-hour shifts (18 pre-intervention and 9 post intervention with Ward Assistants), a mix of night and day shifts. Table 3. A total of seven FGDs were conducted with a total of 40 mothers participating. Additionally, 46 interviews were conducted with the nurses and clinicians. The non-structured observations, (which included aspects of IPC), for the larger study were carried out over 1800 h, carried out in 260 shifts, (150 pre-intervention and 110 post-intervention). Table 3 below provides a summary of the data collection during the study period.

**Table 3:** Outline of hospital data collection

Characteristic		H1		H2		H3		H4		Overall
Hospital Assessments by the research assistants		**Pre intervention**	**Post Intervention**	**Pre intervention**	**Post Intervention**	**Pre intervention**	**Post Intervention**	**Pre intervention**	**Post Intervention**	
	Day	3	2	3	2	3	1	2	1	17
	Night	2	1	2	0	2	1	1	1	10
Interviews with nurses**		5	4	10	3	5	6	6	7	46
IPC Focus Group Discussions^#^		0	2	0	2	0	2	0	1	7
Number of observation shifts		47	27	28	28	27	27	48	28	260
Overall, Hours of non-participant observations*		384	150	240	150	192	150	384	150	1798

# FGDs were conducted in the post-intervention period

* Overall hours observation included the IPC observations

### General ward cleanliness

From our assessments and observations across the two data collection rounds, we found the wards across the four hospitals were reported to be visibly clean. This was visible from the clean floors, walls, and surfaces, and how often we observed the wards were cleaned. In the facilities most of the cots were metal made of wire mesh, painted clean and in good condition. The plastic cots also appeared clean in the four facilities. The bed/cot linen and blankets were also noted to be clean across the facilities. There were changed regularly by the staff and ward assistants. The mothers’ gowns were also noted to be clean, and mothers were provided with a clean pair upon request. Addition data from the FGDs, the mothers also reported noticing the wards were clean.

*“…sanitation is all right; we can’t complain about cleanliness. Even if you look around the whole place is clean*,* even the toilets*,* and other places we can’t complain. Hygiene is okay*,* according to the time I have been here*,* and it’s been almost a month.” (Hospital 3 Mother)*.

### Hand hygiene practices and supplies

#### Mothers hand hygiene

**Fig. 1:**
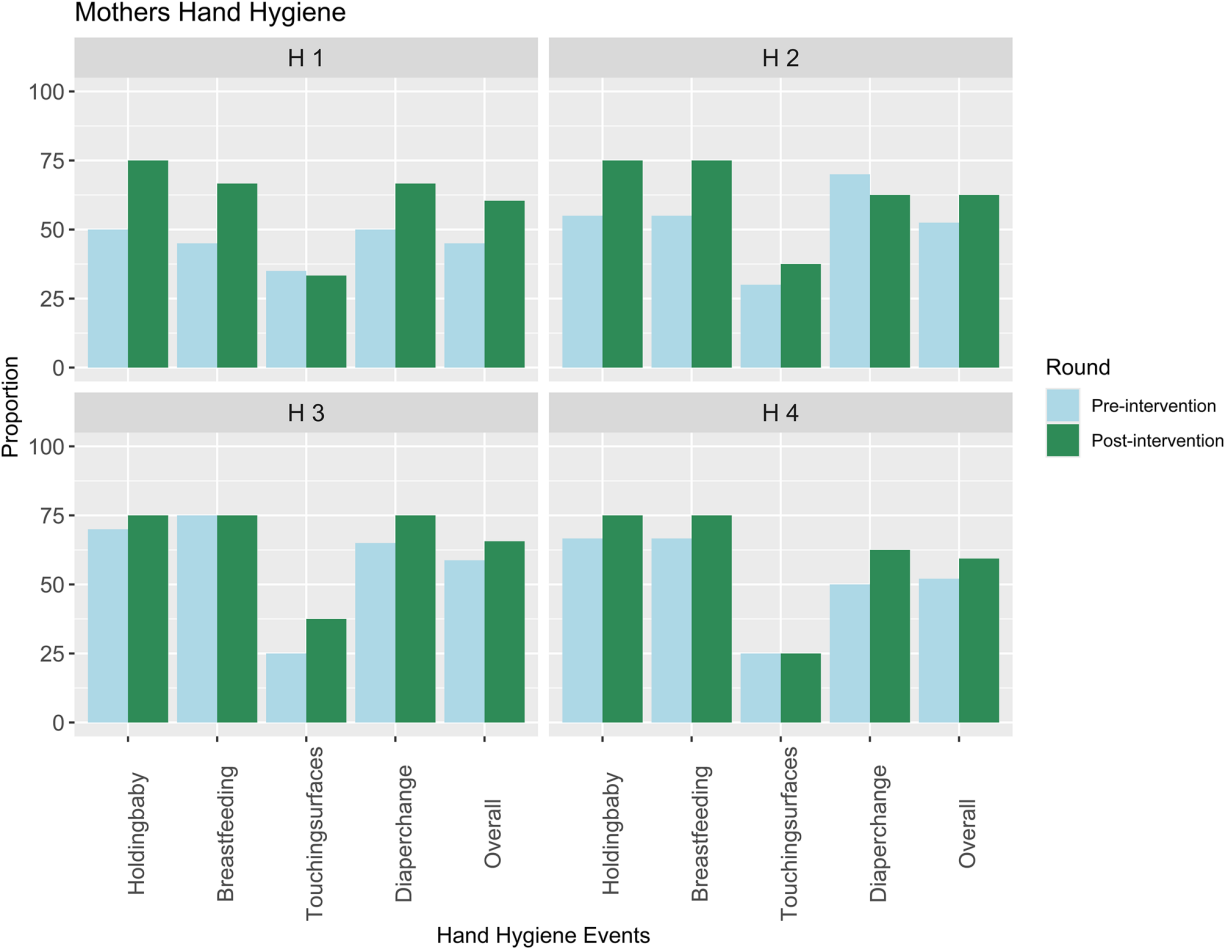
Mothers hand hygiene performance

Mothers’ hand hygiene practices were observed and reported from the structured assessments. Based on the number of babies in the ward per shift, these observer scores were based on observing an average of 30 mothers per shift per hospital during the pre-and post-intervention periods. Overall performance ranged between 45 and 58% in the first round and was slightly higher in the post-intervention cycle at 60–65% across all the hospitals. Hand hygiene was noted to be poorest (< 40% score) after the mothers touched hospital surfaces (Fig. 1).

The findings on hand hygiene reported above are further corroborated by findings from the non-structured observation data which show that some mothers were compliant with observing hand hygiene practices upon their arrival to the newborn units during the designated feeding hours.

*Some mothers wash their hands before coming in*,* but others just walk past the sink without washing.* (Hospital 1 Observer- Field Notes)

However, compliance with hand hygiene practices was not uniform for all mothers across the facilities. Observation data captured some instances where mothers did not wash their hands on their arrival at the NBU and after completion of scheduled tasks during the feeding hours. This is despite having running water and the mothers receiving the necessary training on hand hygiene from the ward staff. However, these instances were uncommon.

*As mothers are coming in at noon*,* I[observer] hear a mother saying she has been told that they need to wash their hands before they come in… Mothers don’t wash their hands inside the changing area yet there is running water.* (Hospital 3 Observer- Field notes)

To improve compliance with hand hygiene practices, the supportive role provided to mothers by healthcare providers including the ward assistants was also noted. This was shown through the orientation of mothers on hand hygiene. Aspects covered during the orientation process included showing them where the sinks for handwashing were located and providing them with information on the need to wash hands using soap and water.

*“So*,* we keep on reminding them of course these mothers you have to remind them all the time because maybe they came from an environment where hand washing is not a must but*,* in the hospital*,* you know now like NBU you have to tell them.”* (Hospital 2 Nurse).

Further, the observation data below provides the context of a healthcare provider supporting a mother visiting the newborn unit for the first time. This support included communication on hand hygiene.

*A new mother stands next to the nurse’s desk. She is stranded and the nurse is busy with the students. She talks to a nurse about her baby. She tells her*,* “I have come from ward xxx and the nurse told me my baby is here.” The nurse asks*,* “When did you deliver and what is your name?” After the mother responds*,* the nurse then leads her to the preterm room and shows her the baby. Before entering the room*,* the nurse shows her the handwashing sink and tells her to wash her hands with the liquid soap placed next to the sink. She also tells her to dry her hands in the acute room using the heaters.* (Hospital 4 Observer Field notes).

#### Nurses, clinicians, and students’ hand hygiene

**Fig. 2:**
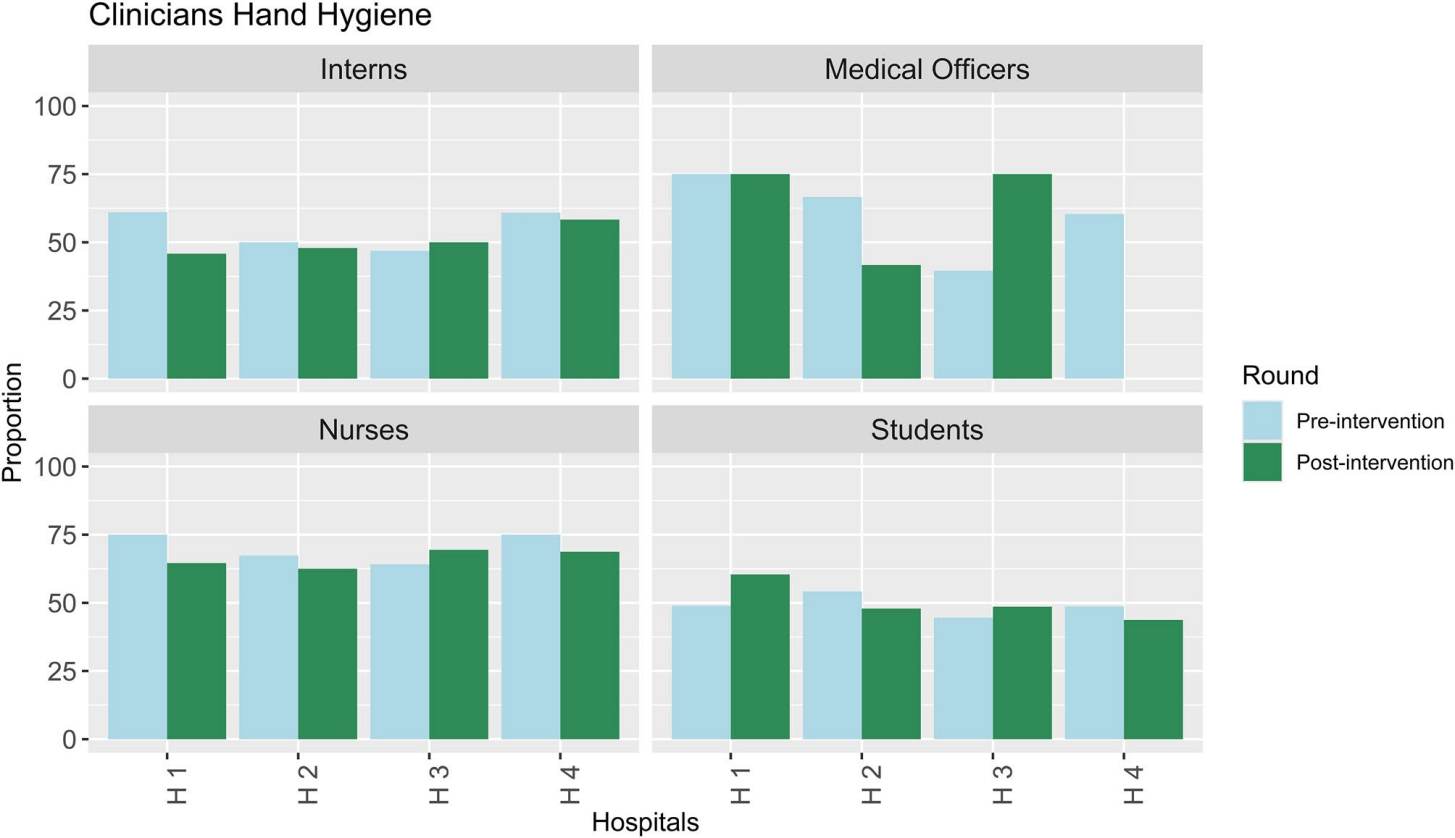
Hand hygiene performance among clinicians and students

Part of the structured assessments included observing the clinicians’ and students’ hand hygiene practices using the five moments of hand hygiene [15]. Overall, nurses had better hand hygiene practices compared to other clinicians. There was poor performance among students, with most facilities scoring less than 50% across the two rounds of data collection for student hand hygiene practices (Fig. 2, in the post-intervention period, no medical officers were observed in H2).

While healthcare providers tried their best to observe IPC, we noted instances where the knowledge on hand hygiene methods was lacking, and some seemed to see the use of alcohol hand rubs as inferior. This points to the need for frequent training on hand hygiene in these units.

*“Well*,* we try to maintain the cleanliness by making sure we clean our hands often with soap and water*,* we have sanitisers [alcohol hand rub] around*,* but I think*,* we don’t do a good job about that because for example*,* if you’re giving medication on this side on the preterm side*,* we rarely go clean our hands. We just use the sanitiser*,* which I think is not okay to use it so many times. But I think at some point we fail*,* but we try at least to sanitize. Yeah. Although I think the frequency of washing our hands should be improved.”* (Hospital 1 Nurse).

Further, from the FGDs the findings reveal lapses in the student nurses’ observing hand hygiene practices. The mothers reported differences in hand hygiene practices between the qualified nurses and those in training.

*“On the nurses’ side*,* it’s alright*,* but for students [student nurses]*,* not all*,* others*,* you find she’s attending to you*,* she doesn’t have gloves*,* and when she’s called*,* she won’t wash her hands*,* she’ll just go there without gloves. Also*,* you find some have gloves and after attending to the child*,* when she’s called*,* she won’t remove the gloves*,* she’ll use the same gloves*,* and she won’t sanitize.” (Hospital 2 Mother)*.

Other than the student nurses, from the interviews, due to the three-monthly rotations of medical interns, there was a general impression of the need to orient clinicians rotating in the units on hand hygiene to enhance IPC.

*“I can say because in NBU we keep having new students*,* nurses and even the MO and clinical officer intern who keep rotating. I would…suggest*,* every time we have a new team*,* if it’s from the nursing side*,* let there be an orientation before*,* especially on infection prevention*,* because for us who are already there*,* we know how to prevent infection…” (Nurse Hospital 3).*

#### Equipment and supplies for IPC

For this domain, all four facilities had hand hygiene stations (some were dysfunctional) and posters. There were adequate supplies of 70% alcohol handrub in three of the facilities with scores ranging from 70 to 100% availability in both rounds of observation. In the other facility this was recorded at 40 and 70% in both assessment rounds. However, water supply availability varied across the facilities and none of the facilities had hand towels next to the sinks. The non-participant observations and interviews also reported data on the availability of hand hygiene equipment and supplies. Interruptions in water supply and sporadic shortages in hand hygiene supplies including the had rubs prompted adaptation measures (“workarounds”) among healthcare providers. Such measures included using methylated spirit which is inappropriate for hand hygiene.

*“Hand washing is not followed satisfactorily and for this*,* it’s quite complex because sometimes you will want to but there is no water*,* other times people will[clean their hands]*,* because if you endeavour to clean your hands you find no water*,* your next resort will be a sanitiser which is not always there*,* so we use methylated spirit which is not so good.”* (Hospital 3 Medical Officer).

Additional information on the equipment and supplies for IPC has been included in a figure in the supplementary materials (Appendix 5).

### Waste management

From the observations and assessments, the sharps box, and the colour-coded bins were present in the hospitals. Waste was also segregated into the appropriate bins. However, waste disposal pictures with segregation instructions were not available at all the waste disposal sites within the units. This brought the score for this indicator to 40–70% across the facilities.

### Cleaning of hospital equipment

The structured assessments observed the cleaning and decontamination of selected hospital equipment. In these facilities, none of the devices had cleaning rotas or cleaning instructions available. This brought the overall performance for this domain to less than 30% for all the hospitals in the two rounds of observation. Across the hospitals, we noted different compounds were used to clean the equipment. These included a common chlorine-based household cleaner and disinfectant “Jik^®^”, for cleaning equipment like incubators.

*“We have the incubator; you don’t use spirit. That one has its special cleaning agent*,* so that one I must use it. If maybe it’s out of stock*,* I use plain water or chlorinated water*,* maybe this portion of Jik and this portion of water*,* then I clean. That is for the incubator. Yeah.”* (Hospital 4 Nurse).

High demand for the limited equipment affected their cleaning and decontamination. For instance, since there was a high demand for incubators, we observed inadequate cleaning where some of them were cleaned on the outside and the inside was left uncleaned.

*“We always use Jik*,* as our major cleaning…sometimes we don’t have the Jik*,* you know that but what is expected of us especially if we have students [student nurses]*,* if the incubators have no babies*,* we are supposed to dismantle [disassemble] it. We are supposed to remove it from the room bring it to this side so that we dismantle [disassemble] it and then we clean with Jik*,* leave it to air dry and then we return it but*,* in most cases*,* when we have so many babies*,* we may not dismantle[disassemble] it. You’ll clean it on the side. You just put some Jik with some clean cloth or cotton wool*,* just decontaminate it*,* air dry it and put another baby.”* (Hospital 3 Nurse).

Equipment sharing of some equipment like incubators and phototherapy machines affected how the equipment was cleaned.

*“Again*,* it is another challenge because like the new phototherapies we have*,* ideally*,* they are supposed to be used for one baby*,* but we get so many babies who require that phototherapy*,* so they end up sharing.*,* After you remove one*,* you find you already having another baby who requires that. So*,* you use the alcohol. We swab and we use it again.”* (Nurse Hospital 3).

From the FGDs mothers expressed discomfort with their babies’ sharing incubators and, in some instances, the mothers cleaned the incubators themselves.

*“The cleaning of the incubator*,* I have a problem with that*,* because like now the baby in our incubator has been vomiting for the last three days. And you can see the stains of the vomit*,* and we’ve been the ones [mothers] doing the cleaning [wiping]ourselves*,* so*,* I just wipe where my baby is*,* and when I ask her to clean*,* she is a bit apprehensive*,* she perceives as if I’m instructing her on what she should do.* (Hospital 1 Mother)

### Cleaning of single-use equipment

Across the facilities, healthcare providers experienced challenges with consumable items such as nasal prongs, and CPAP connection tubes. Due to their low supply and high demand, these items were reprocessed using soap, chlorine and water and reused. These tasks were shared between the nurses and the ward assistants.

*“Let’s say for example a baby comes and needs NRM [nonrebreather mask]. I’m supposed to wash it before inserting. The tubes are being washed by our casual [ward assistant] with Jik there is a drum there for that. They are trying to wash. We have instructed them*,* once they have soaked in that Jik*,* let them rinse in the water.”* (Hospital 2 Nurse).

### Effects of ward assistants

As described above the mothers from the FGDs reported the wards were generally clean, much of which can be attributed to the ward assistants. The effects of the ward assistants on IPC were however noted in two major domains (ward cleanliness and waste management). Here we present data from our interviews on the effects of ward assistants on IPC.

#### General cleanliness

From the interviews, improvements were reported following the introduction of ward assistants this included the number of times the wards were cleaned.

*“…we have seen a great improvement on it*,* working condition*,* the environment is clean*,* that’s one…before the ward assistants came in*,* the wards could have not been clean as compared today. I think we have seen some great improvements.”* (Hospital 1 Nurse).

#### Waste management

From the interviews, the improvements in waste management were reported to have resulted from the increase in the number of ward assistants in the newborn units (after the intervention). Waste management was one of the tasks that had been assigned to the ward assistants in the units.

*“Yes*,* there is a change. Because before[ward assistants’ intervention]you might find maybe these bins you could find some are full. They have not been emptied and maybe when you inquired the ward assistant who was there*,* she was only one and she had a lot of work and has not had enough time to be able to clear them out. But since the introduction [ward assistants] I haven’t seen such a scenario again. I think they have been able to deal with the waste properly.”* (Hospital 1 Nurse).

## Discussion

Improving IPC is critical in improving the quality of neonatal care. The WHO Maternal and Newborn Care quality standards speak to the importance of reliable and functional water, energy, sanitation, hand hygiene and waste disposal facilities [19]. It was against these standards we describe our findings in this report. Poor IPC structures not only increase the length of hospital stay, due to hospital-acquired infections but also increase the costs of providing care and end up increasing the workload for an already stretched workforce [6].

In addition to the provision of equipment in these hospitals, the NEST 360 programme provided training and instructions on care and maintenance, including the cleaning and decontamination of the equipment [5]. During the study, these instructions and device cleaning registers/logs were missing, and some equipment was visibly soiled. Due to the high patient loads, device cleaning and decontamination were reported to be a challenge. High patient loads and device sharing including incubators and radiant warmers have been described as key drivers for healthcare-associated infections in neonatal units [20]. The reuse of single-use, semi-critical devices (contact with mucous membranes); and non-critical devices (contact with unbroken skin) such as nasal prongs and face masks is common in many hospitals in resource-limited settings and was also witnessed in this study. Single use may not be feasible due to resource constraints and therefore items tend to be disinfected and reused against the manufacturer’s recommendation. Manufacturers should consider items that can be used multiple times and provide clear reprocessing instructions and procedures. The number of times a device can be reprocessed should also be documented [21].

Erratic supplies of IPC-related materials and equipment have a profound effect on IPC [22]. The hospitals reported frequent water shortages, the absence of alcohol hand rubs, paper towels and materials for cleaning equipment like incubators. This makes it difficult to reinforce good practices among the healthcare workers, students, and mothers. As a result, adaptive improvisations such as using methylated spirit for hand hygiene and chlorine-based solutions to clean equipment were observed.

We noted in this study the healthcare providers perceived the use of alcohol had rubs as inferior to using soap and water for hand hygiene. Additionally, we recorded instances where mothers and caregivers walked past the hand hygiene stations without using them. These gaps point to the need to determine the training needs/gaps of the healthcare providers and caregivers regarding IPC and tailor targeted interventions or training modules that seek to address these gaps for continuous improvement.

However, these improvisations are both ineffective and damaging to medical equipment. Staff shortages and workload are also a key constraint for practicing infection prevention and hand hygiene. In units where these staff members have numerous competing tasks, these IPC related tasks may not be prioritised in the face of other more pressing (immediate) tasks, such as are needed to tend to the immediate care needs of very sick neonates [23, 24].

After the introduction of the ward assistants, we noted an improvement in key IPC areas, notably ward cleanliness and waste management. Some of these tasks like cleaning and waste management were directly included in their job descriptions and hence we expected notable improvement in these domains. Other key tasks that the ward assistants carried out under the supervision of nurses included cleaning (medical) equipment in the neonatal units. The introduction of ward assistants was meant to free up the time for nurses and provide enhanced support, allowing nurses to focus more on patient care including monitoring, treatment, and counselling and enhancing family-centred care [25, 26].

We argue that investing in extra workforce to assist nurses in these facilities improves the IPC status of the facility. Our data show that sometimes the facilities we observed were lacking essential IPC resources such as consistent running water, hand soap and sanitisers, and equipment disinfectant. However, we also found instances where the resident clinicians and nurses were not observing current IPC procedures despite having the requisite training and resources. Healthcare worker behaviours and lack of patient safety cultures in the neonatal units in resource-limited settings have contributed to poor IPC practices in these hospitals. Enhancing this culture, which encompasses individual and collective actions and attitudes in a setting where the individuals are knowledgeable and competent on IPC matters is critical to improving IPC [27].

In summary, improving IPC and hand hygiene in neonatal units low-resource settings requires a multifaceted approach that encompasses more consistent and adequate supplies including the appropriate equipment cleaning agents, more professional nursing staff in general and additional human resources like ward assistants to support, promote and take on less technical roles including educating/reinforcing behaviours of mothers [28, 29]. The health workforce have a responsibility to adhere to safe practices like hand hygiene compliance [30].

We acknowledge this study has some limitations; structured assessments using checklists, and our rating system, were subjective. This was however mitigated by training our observers and piloting the tools before data collection. Additionally, our observational findings were corroborated with data from our interviews and focus group discussions; we saw congruence across our measures, which increases robustness of our findings. We acknowledge that project staff conducting the interviews were linked to the implementing team and had the potential to introduce bias in interviews. However, this was minimized by non-participant observations in the NBUs and FGDs with mothers to triangulate interview data.

We propose future work in this area should seek the use of novel tools like fluorescent markers, colony counts and adenosine triphosphate (ATP) assays to quantitatively measure ward cleanliness [31]. Additional work to evaluate the clinical outcomes and compare infection rates with such interventions would bolster the evidence in this area.

## Conclusion

The addition of ward assistants into neonatal units was noted to provide an improvement in key IPC domains including general cleanliness, waste handling and hand hygiene for the carers. These positive effects were however marred by the erratic supply of crucial IPC materials. To realise a sustained improvement in IPC, the improvement of human resources and sustained supplies needs to be accompanied by a change in patient safety attitude/culture by the health workers.

## Electronic supplementary material

Below is the link to the electronic supplementary material.

Supplementary Material 1

## Data Availability

The data used for this report is available upon request. Applications for access can be made through the Data Governance Committee on email to cgmrc@kemri-wellcome.org.

## Abbreviations

CPAP: Continuous positive airway pressure machines
FGD: Focus group discussion
HCAI: Healthcare-associated infections
IPC: Infection prevention and control
LMIC: Low-and middle-income countries
NBU: Newborn unit
WHO: World health organization
