# Supplementary material for: Strengthening neonatal care through ward assistants: a Kenyan case study in enhancing infection prevention and control practices
Source: Antimicrob Resist Infect Control. 2025 Jun 2;14:61. doi: 10.1186/s13756-025-01575-w (PMC12131407; doi:10.1186/s13756-025-01575-w)

|  |  | **Hospital** | | | |
| --- | --- | --- | --- | --- | --- |
|  |  | **H1** | **H2** | **H3** | **H4** |
| **Location of the Hospital** |  | Urban | Urban | Semi-urban | Semi-urban |
| **NBU Bed Capacity** |  | 55 | 60 | 50 | 38 |
| **Average bed occupancy** |  | 107% | 72% | 54% | 50% |
| **Number of babies on the ward (median IQR)** |  | 59 [53, 64] | 43 [39, 47] | 27 [24, 31] | 19 [16, 21] |
| **Number of nurses on the Shift (median IQR)** | Day | 2 [2-3] | 2 [2-3] | 1 [1-2] | 2 [1-2] |
|  | Night | 2 [2-2] | 3 | 1 [1-2] | 1 [1-2] |
| **Nurse-to-babies ratio** |  | 1:37 (34-43) | 1:20 (20-23) | 1:23 (17-30) | 1:15 (12-18) |
| **Total Workforce** | **Skill mix** |  |  |  |  |
| **Nurses** | Certificate | 1 (7%) | 1 (6%) | 1 (17%) | 0 |
|  | Diploma | 8 (57%) | 11 (61%) | 5 (83%) | 7 (58%) |
|  | Higher Diploma (Neonatal Care) | 4 (29%) | 3 (17%) | 0 | 1 (8%) |
|  | Bachelor’s degree | 1 (7%) | 3 (17%) | 0 | 4 (33%) |
| **Paediatrician/Neonatologist** | Consultant | 2 | 2 | 1 | 2 |
| **Medical Officers**  **(General practitioner)** | Medical Officer | 1 | 0 | 1 | 1 |
|  | Medical Officer Interns | 3 | 2 | 2 | 1 |
| **Clinical Officers Non (Physician clinicians)** | Clinical Officer Interns | 1 to 5 | 0 to 5 | 2 to 5 | 1 to 4 |
| **Nutritionist** | Nutritionist | 1-2 | 1 | 1 | 1 |
| **Nursing Students**, **median [IQR]** | Student nurses | 2 [1-5] | 7 [4-12] | 7 [4-14] | 6 [3-11] |
| **Ward Assistants (WA)** | WA Pre-intervention | 3 | 3 | 1 | 2 |
|  | WA post-intervention | 6 | 5 | 4 | 5 |

Appendix 1 Hospital Characteristics and Workforce

Appendix 2 Quantitative Data collection Tool

| **SECTION** | | **QUESTION** | | **COMMENTS** |
| --- | --- | --- | --- | --- |
| **A** | Hygiene in the newborn unit | 1 | On what occasions do mothers/caregivers wash their hands?   1. before holding the baby   1 Never 2 Rarely 3 Occasionally 4 Often 5 Always |  |
|  |  |  | 1. before breastfeeding/feeding the baby   1 Never 2 Rarely 3 Occasionally 4 Often 5 Always |  |
|  |  |  | 1. after changing the baby’s diaper/nappy   1 Never 2 Rarely 3 Occasionally 4 Often 5 Always |  |
|  |  |  | 1. after touching hospital surfaces or equipment   1 Never 2 Rarely 3 Occasionally 4 Often 5 Always |  |
|  |  |  | 1. Other? (Describe the other besides I-IV above)   1 Never 2 Rarely 3 Occasionally 4 Often 5 Always 6. None/Not Applicable |  |
|  |  |  | 1. Baby feeding cups/syringes are cleaned through a decontamination process   1 Never 2 Rarely 3 Occasionally 4 Often 5 Always |  |
|  |  |  | 1. Baby nasal prongs; Oxygen/suction tubes; pigeon suckers/aspirators are cleaned through a decontamination process   1 Never 2 Rarely 3 Occasionally 4 Often 5 Always |  |
|  |  | 2 | Is there continuous supply of water and soap for handwashing in or around the wards?  1. Yes 2. No |  |
|  |  | 3 | Is there continuous supply of single use hand towels at the handwashing station?  1. Yes 2. No |  |
|  |  | 4 | Are sinks for handwashing available and what is the cleanliness and functionality status?  1. Yes 2. No |  |
|  |  | 5 | Is there continuous supply of hand sanitizer in the ward?  1. Yes 2. No |  |
|  |  | 6 | Are there posters/charts on handwashing in the ward or hand washing stations? Proportion of sinks with hand hygiene materials on the wall?  1. Yes 2. No |  |
|  |  | 7 | Describe the cleanliness status of ward walls and floors:   \|  \|  \| YES \| NO \| \| --- \| --- \| --- \| --- \| \| a) \| Walls are visibly clean \|  \|  \| \| b) \| Walls are visibly dusty/dirty \|  \|  \| \| c) \| Painting on walls appear clean/fresh \|  \|  \| \| d) \| Floors and horizontal work surfaces appear clean \|  \|  \| \| e) \| Some floors and work surfaces appear clean, but others do not \|  \|  \| \| f) \| Most floors and work surfaces are visibly dirty \|  \|  \| |  |
|  |  | 8 | Describe the cleanliness status of beds and cots |  |
|  |  | 9 | Describe the cleanliness of changing bed linen & mothers’ gowns |  |
|  |  | 10 | Describe the cleanliness of the healthcare provider gowns/scrubs/coats |  |
|  |  | 11 | Describe the adequacy and cleanliness of Crocs/Sandals for both staff and mothers |  |
| **B** | 1. Clinical staff hygiene - **Nurses** | 12 | Are nurses performing hand hygiene before touching each baby? e.g., hand washing, alcohol hand rub or use of gloves  1 Never 2 Rarely 3 Occasionally 4 Often 5 Always |  |
|  |  | 13 | Are nurses hand washing or sanitizing before wearing a new pair of gloves?  (*This is before a clean or aseptic procedure*)  1 Never 2 Rarely 3 Occasionally 4 Often 5 Always |  |
|  |  | 14 | Are nurses wearing a new pair of gloves before conducting procedures e.g., drawing blood, inserting NG tube?  1 Never 2 Rarely 3 Occasionally 4 Often 5 Always |  |
|  |  | 15 | Are nurses hand washing or sanitizing their hands after touching a patient?  1 Never 2 Rarely 3 Occasionally 4 Often 5 Always |  |
|  |  | 16 | Are nurses hand washing after contact with patients’ body fluids?  1 Never 2 Rarely 3 Occasionally 4 Often 5 Always |  |
|  |  | 17 | Are nurses hand washing or sanitizing their hands after contact with a patient’s surrounding?   1. Never 2 Rarely 3 Occasionally 4 Often 5 Always |  |
|  | 1. Clinical staff hygiene - **Clinical Officers** | 18 | Are Clinical officers performing hand hygiene before touching each baby? e.g., hand washing, alcohol had rub or use of gloves  1 Never 2 Rarely 3 Occasionally 4 Often 5 Always |  |
|  |  | 19 | Are Clinical officers hand washing or sanitizing before wearing a new pair of gloves?  1 Never 2 Rarely 3 Occasionally 4 Often 5 Always |  |
|  |  | 20 | Are clinical staff wearing a new pair of gloves before conducting aseptic procedures e.g., drawing blood, giving injections?  1 Never 2 Rarely 3 Occasionally 4 Often 5 Always |  |
|  |  | 21 | Are Clinical officers hand washing or sanitizing their hands after touching a patient?  1 Never 2 Rarely 3 Occasionally 4 Often 5 Always |  |
|  |  | 22 | Are Clinical officers hand washing after contact with patients’ body fluids?  1 Never 2 Rarely 3 Occasionally 4 Often 5 Always |  |
|  |  | 23 | Are Clinical officers hand washing or sanitizing their hands after contact with a patient’s surrounding?   1. Never 2 Rarely 3 Occasionally 4 Often 5 Always |  |
|  | 1. Clinical staff hygiene - **Medical Officers** | 24 | Are doctors performing hand hygiene before touching each baby? e.g., hand washing, alcohol had rub or use of gloves  1 Never 2 Rarely 3 Occasionally 4 Often 5 Always |  |
|  |  | 25 | Are doctors hand washing or sanitizing before wearing a new pair of gloves?  1 Never 2 Rarely 3 Occasionally 4 Often 5 Always |  |
|  |  | 26 | Are doctors wearing a new pair of gloves before conducting aseptic procedures e.g., drawing blood, giving injections?  1 Never 2 Rarely 3 Occasionally 4 Often 5 Always |  |
|  |  | 27 | Are doctors hand washing or sanitizing their hands after touching a patient?  1 Never 2 Rarely 3 Occasionally 4 Often 5 Always |  |
|  |  | 28 | Are doctors hand washing after contact with patients’ body fluids?  1 Never 2 Rarely 3 Occasionally 4 Often 5 Always |  |
|  |  | 29 | Are doctors hand washing or sanitizing their hands after contact with a patient’s surrounding?  1 Never 2 Rarely 3 Occasionally 4 Often 5 Always |  |
|  | 1. Interns (MO/CO/NO) | 30 | Are interns performing hand hygiene before touching each baby? e.g., hand washing, alcohol had rub or use of gloves  1 Never 2 Rarely 3 Occasionally 4 Often 5 Always |  |
|  |  | 31 | Are interns hand washing or sanitizing before wearing a new pair of gloves?  1 Never 2 Rarely 3 Occasionally 4 Often 5 Always |  |
|  |  | 32 | Are interns wearing a new pair of gloves before conducting aseptic procedures e.g., drawing blood, giving injections?  1 Never 2 Rarely 3 Occasionally 4 Often 5 Always |  |
|  |  | 33 | Are interns hand washing or sanitizing their hands after touching a patient?  1 Never 2 Rarely 3 Occasionally 4 Often 5 Always |  |
|  |  | 34 | Are interns hand washing after contact with patients’ body fluids?  1 Never 2 Rarely 3 Occasionally 4 Often 5 Always |  |
|  |  | 35 | Are interns hand washing or sanitizing their hands after contact with a patient’s surrounding?  1 Never 2 Rarely 3 Occasionally 4 Often 5 Always |  |
|  | 1. **Students** | 36 | Are students performing hand hygiene before touching each baby? e.g., hand washing, alcohol had rub or use of gloves  1 Never 2 Rarely 3 Occasionally 4 Often 5 Always |  |
|  |  | 37 | Are students hand washing or sanitizing before wearing a new pair of gloves?  1 Never 2 Rarely 3 Occasionally 4 Often 5 Always |  |
|  |  | 38 | Are students wearing a new pair of gloves before conducting aseptic procedures e.g., drawing blood, giving injections?  1 Never 2 Rarely 3 Occasionally 4 Often 5 Always |  |
|  |  | 39 | Are students hand washing or sanitizing their hands after touching a patient?  1 Never 2 Rarely 3 Occasionally 4 Often 5 Always |  |
|  |  | 40 | Are students hand washing after contact with patients’ body fluids?  1 Never 2 Rarely 3 Occasionally 4 Often 5 Always |  |
|  |  | 41 | Are students hand washing or sanitizing their hands after contact with a patient’s surrounding?  1 Never 2 Rarely 3 Occasionally 4 Often 5 Always |  |
| **C** | Covid-19 prevention measures  **(Describe)** | 42 | Wearing face masks  1 Never 2 Rarely 3 Occasionally 4 Often 5 Always |  |
|  |  | 43 | Social distancing  1 Never 2 Rarely 3 Occasionally 4 Often 5 Always |  |
|  |  | 44 | Limiting number of visitors (inquire about unit rules on visitors)  1 Never 2 Rarely 3 Occasionally 4 Often 5 Always |  |
|  |  | 45 | No handshaking  1 Never 2 Rarely 3 Occasionally 4 Often 5 Always |  |
|  |  | 46 | Availability of posters and other information, communication, and education materials on Covid-19 in the newborn ward  1. Yes 2. No |  |
|  |  | 47 | Sanitizing/cleaning of commonly touched surfaces e.g., door handles, tables, trolleys, bed rails and frames  1 Never 2 Rarely 3 Occasionally 4 Often 5 Always |  |
| **D** | Equipment use | 48 | **Contact thermometer** (should be cleaned with alcohol before /after every patient use) For contact thermometers, record the proportion of babies where the thermometer was cleaned before use.  **NOTE:** IF THEY USE THERMOGUNS CLEARLY STATE SO |  |
|  |  | 49 | **Stethoscope:** It should be cleaned with alcohol before /after every patient use. Record the proportion of babies where the stethoscope was cleaned before use. |  |
|  |  | 50 | **Pulse oximeter:** It should be cleaned with alcohol before /after every patient use). Record the proportion of babies where the POX was cleaned before use. |  |
|  |  | 51 | **Incubators:**  Is a cleaning rota available? 1. Yes 2. No  Is there an instruction sheet stating how the equipment should be cleaned?  1. Yes 2. No  While in the ward, were the incubators cleaned?  1. Yes 2. No |  |
|  |  | 52 | **Continuous positive airway pressure machine (CPAP):**  Is a cleaning rota available? 1. Yes 2. No  Is there an instruction sheet stating how the equipment should be cleaned?  1. Yes 2. No  While in the ward, were the CPAP machines cleaned?  1. Yes 2. No |  |
|  |  | 53 | **Oxygen concentrator:**  Is a cleaning rota available? 1 Yes 2 No  Is there an instruction sheet stating how the machine should be cleaned? 1 Yes 2 No  While in the ward, was the machine cleaned? 1 Yes 2 No |  |
|  |  | 54 | **Phototherapy device:**  Is a cleaning rota available? 1 Yes 2 No  Is there an instruction sheet stating how the machine should be cleaned? 1 Yes 2 No  While in the ward, were the devices cleaned? 1 Yes 2 No |  |
|  |  | 55 | **Radiant warmer:**  Is the cleaning rota available? 1 Yes 2 No  Is there an instruction sheet stating how the machine should be cleaned? 1 Yes 2 No  While in the ward, were radiant warmers cleaned?  1 Yes 2 No |  |
| **E** | Equipment sharing | 56 | Are babies sharing cots with other babies?  1 Yes 2 No |  |
|  |  | 57 | Are babies sharing incubators?  1 Yes 2 No |  |
|  |  | 58 | Are babies sharing phototherapy devices?  1 Yes 2 No |  |
|  |  | 59 | Are babies sharing radiant warmers?  1 Yes 2 No |  |
| **F** | Waste disposal  **CODE**  **Black** - Non-infectious waste  **Yellow** - Infectious waste  **Red** - Highly infectious waste | 60 | Are waste segregation bins **available** and **correctly labelled**?   \|  \|  \| YES \| NO \| \| --- \| --- \| --- \| --- \| \| a) \| Non-Infectious/General waste (Black) Bin \|  \|  \| \| b) \| Infectious waste (Yellow) Bin \|  \|  \| \| c) \| Highly Infectious waste (Red) Bin \|  \|  \| \| e) \| Sharps box/containers \|  \|  \| \| f) \| Other bin(s) present (*describe*) \|  \|  \| \|  \| Specify other bins (above) ………………………………………………… \| \| \| |  |
|  |  | 61 | Presence of functional waste collection bins:   \|  \|  \| YES \| NO \| \| --- \| --- \| --- \| --- \| \| a) \| Is waste is correctly segregated in the bins? (*check the bins*) waste \|  \|  \| \| d) \| At least one of the above waste bins is > **¾** full \|  \|  \| \| e) \| Sharps box/containers are near waste generation points) \|  \|  \| \| f) \| Sharps box/container is > **¾** full \|  \|  \| \| g) \| Are pictures of waste segregation available next to the waste collection bins? \|  \|  \| |  |
| **G** | Decontamination of equipment and devices | 64 | 1. Decontamination buckets present in the unit:  \|  \| YES \| NO \| \| --- \| --- \| --- \| \| 1. Chlorine solution \|  \|  \| \| 1. Soapy water \|  \|  \| \| 1. Clean (rinsing water) \|  \|  \| \| 1. The above decontamination buckets were prepared today \|  \|  \| |  |
|  |  | 65 | Describe the cleanliness status of the basins/bowls used in top-tailing/cleaning the baby. |  |
|  |  | 66 | Describe how the cleaning of basins/bowls used to top-tail the baby is done. |  |

Appendix 3

**Appendix 3 Interview Guides**

**Healthcare Providers IPC Interview Guide**

1. In the ward, what is the cleaning routine for the following equipment: Incubators, CPAP, Oxygen concentrator, phototherapy devices and radiant warmers? When more than one baby is utilizing the machines, how is cleaning managed?
2. How would you describe your experience with cleaning medical equipment in the neonatal ward? (Probe: What are the challenges you have been facing with cleaning medical equipment e.g. some equipment being more difficult to clean than others, availability of cleaning materials, cleaning materials being abrasive to the equipment, time to clean, cleaning know how, cleaning norms in the ward? What are the possible effects caused by these challenges? Have any measures been put in place to address these challenges and are they working?)
3. How would you describe patient/caregivers’ or mothers’ sanitation and hygiene experience while at the newborn unit? (Probe for 1) Availability of water, soap, handwashing sinks, sanitizer 2) hand hygiene (handwashing and sanitizing) 3) baby cots/ beds 4) mothers/caregivers sleeping area 5) other hospital equipment used by their baby 6) walls 7) floors 8) bathrooms 9) toilets 10) How about the availability of waste disposal bins?
4. For the patients/mothers and caregivers in the ward, do you provide any training on handwashing, hand sanitizing and waste disposal according to bin colours?
5. Does cleanliness/hygiene help in a baby’s recovery? How? (Probe: The mother’s/caregiver’s cleanliness, the baby’s cleanliness, the hospital environment’s cleanliness)
6. As a clinician, how would you describe the cleanliness levels of mothers/caregivers? (Probe: Do you feel that you can remind them if notice low cleanliness levels/poor cleanliness practices among them and members of their family? How? Is there anything mothers/caregivers can do to improve cleanliness levels in the newborn unit/ward?)
7. As a clinician, how would you describe the cleanliness levels of clinical staff in the newborn unit? (Probe: Do they always wear fresh pairs of gloves every time before touching each baby? Do they sanitize their hands before or after handling each baby? Is handwashing a common practice among clinicians? From your observations, do clinicians clean thermometers and stethoscopes before or after contact with a baby? Do you feel that you can challenge low cleanliness levels/poor cleanliness practices among clinical staff? How? Is there anything as clinical staff you can do to improve hygiene levels in the newborn unit?)
8. Are any instructions given to caregivers/mothers about covid-19 prevention measures in the wards? (Probe: Have they been able take any preventive measures while in the ward? Which ones and how easy was it to apply them – no hand shaking, handwashing/sanitizing, use of face masks, social distancing, limiting number of visitors, taking a Covid-19 vaccine?)
9. If you would compare the period before the Covid-19 pandemic and now, are there any differences in the hygiene and sanitation practices of mothers and health workers? If yes, which differences are these?

**Caregivers/Mothers IPC FGD Guide**

1. How would you describe your sanitation and hygiene experience while at the hospital? (Probe for a) Availability of water, soap, clean and functional handwashing sinks, sanitizer b) hand hygiene - handwashing and sanitizing c) baby cots/beds d) mothers/caregivers sleeping area e) mothers’ gowns and babies’ linen f) other hospital equipment used by their baby e.g., thermometers, stethoscopes g) walls h) floors i) bathrooms j) toilets k) How about the availability of waste disposal bins?
2. While in the ward, did you receive any training on handwashing, hand sanitizing and waste disposal according to bin colours? If yes, did you learn from these trainings? Have you been able to use the knowledge acquired? If not, why?
3. Did you or your baby share a bed/cot/incubator with any other mother/baby while in the ward? (Probe: How did this make you feel a) sharing a bed with another mother b) your baby sharing a cot or incubator with another? Do you have any concerns with bed/cot/incubator sharing at all?)
4. As caregivers, have you been involved in any hygiene roles in the wards? Probe for cleaning baby feeding items - cups, spoons and syringes, cleaning cots and incubators, and cleaning mothers’ gowns and babies’ linen. How often do they do the roles, how does this task sharing make mothers feel?
5. Does cleanliness help in your baby’s recovery? How? (Probe: The mother’s/caregiver’s cleanliness, the baby’s cleanliness, the hospital environment’s cleanliness)
6. As a mother/caregiver, how would you describe the cleanliness levels of other mothers/caregivers? (Probe: Do you feel that you can remind any of the mothers if you notice low cleanliness levels/poor cleanliness practices among them b) How does a shortage of mothers’ gowns affect hygiene in neonatal units? c) Is there anything as mothers/caregivers you can do to improve cleanliness levels in the newborn unit/ward?
7. How do mothers navigate the need for sanitary pads? Are pads offered by the hospital or do mothers have to buy for themselves? How does this affect mothers’ hygiene?)
8. As a mother/caregiver, how would you describe the cleanliness levels of clinical staff? (Probe: Do they wear fresh pairs of gloves or wash their hands or sanitize their hands every time before touching your baby? Do you feel that you can remind them to do this? How? Is there anything as mothers/caregivers you can do to improve cleanliness levels in clinical staff?)
9. Were any instructions given to you about covid-19 prevention measures in the wards? (Probe: Have you been able take any preventive measures while in the ward? Which ones and how easy was it to apply them – no hand shaking, handwashing/sanitizing, use of face masks, social distancing, limiting number of visitors, taking a Covid-19 vaccine?)
10. Overall, how would you compare your hygiene experience in this health facility with other health facilities you have been to?

Appendix 4 : Ward Assistants Job Description


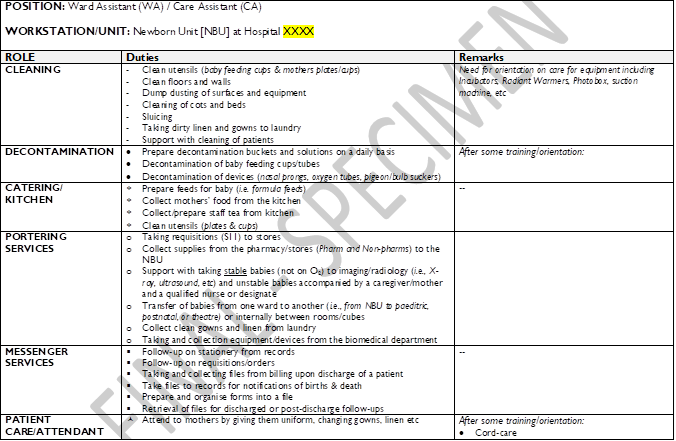


Appendix 5: Figure on Hand Hygiene supplies


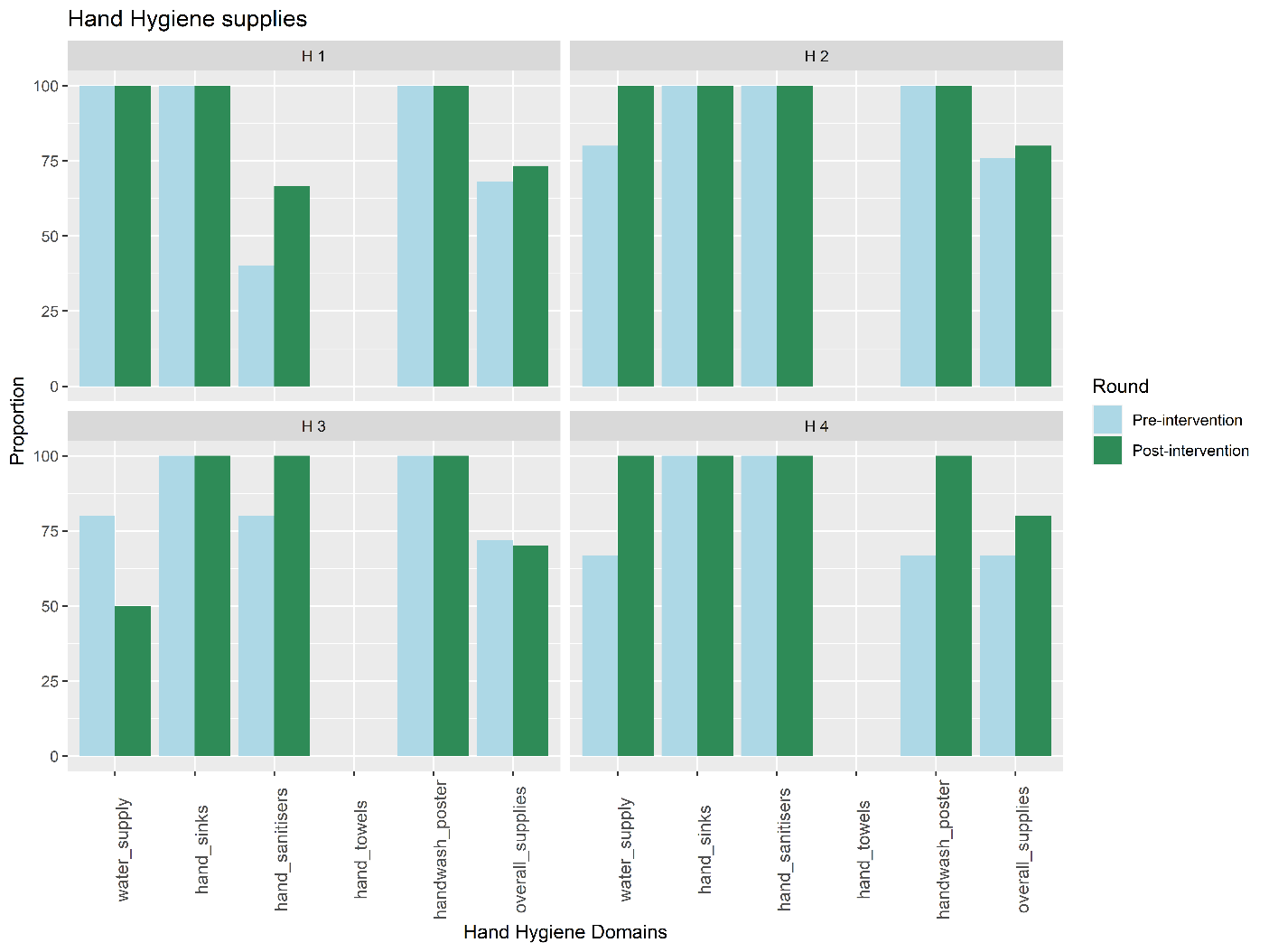

Supplement: Supplementary file 1 — Supplementary Material 1 [file 13756_2025_1575_MOESM1_ESM.docx]
